# Facing migration under adverse conditions: challenges and resilience in the Colombo-Venezuelan border area

**DOI:** 10.1186/s12889-024-21222-0

**Published:** 2025-01-22

**Authors:** Gloria Omaira Bautista, Axel Kroeger, Nelly Rosero Castillo, Eduardo Gabriel Osorio Sanchez, Dianne Sofía Gonzales Escobar, Rafael Olarte, Sonia Diaz Monsalve

**Affiliations:** 1 Departamento Promoción, Departamento Promoción, Protección y Gestión en Salud, Cúcuta, Colombia; 2 Centre for Medicine and Society, University of Freiburg, Freiburg, Germany; 3 Universidad Popular del Cesar, Valledupar, Colombia; 4 Departamento de Derecho y Ciencias Políticas, Universidad Francisco de Paula Santander, Cúcuta, Colombia; 5 Hospital Universitario Erasmo Meoz, Cucuta, Colombia

**Keywords:** Migration, Receptors, Colombia, Venezuela, Mental health, Legal, Health, Violence

## Abstract

**Background:**

Internal displacement and cross-country migration are an increasing global phenomenon drawing the attention of politicians and the public. Causes and effects on the migrants and receptor populations are varied and often shaped by immigration laws and how migrants and refugees are being dealt with by local conditions, policy frameworks and by the host population (receptors). The massive influx of Venezuelan migrants into Colombia for more than a decade has characteristics which warrant a systematic analysis to identify contextual and individual factors favouring and hindering the well-being of migrants and their new Colombian neighbours of the receptor population.

**Methods:**

A household interview survey was conducted in two cities of the Colombo-Venezuelan border: Cucuta and Valledupar. The survey included 412 migrant families, comprising 1656 individuals, and 317 Colombian neighbour families, totalling 1077 individuals. Only migrants staying in the border area for more than 6 months and excluding “short term migrants” who cross the border only for getting treatment or schooling for their children. We used systematic sampling in neighbourhoods with the highest concentration of migrants. The sampling interval was set at every 7 and 6 migrant households. Ethical approval was obtained by the ethics committees of the three participating universities and the local health authority.

**Results:**

Venezuelan migrants were younger than their Colombian neighbours (22.04 and 28.69 years respectively). The primary reason for migration among these individuals was economic (84.1%) and much less for political reasons (< 10%); about half of them had entered the country through an irregular crossings, known locally as “trochas”. Within this group, around 28% reported experiencing various forms of violence, particularly physical or verbal aggression, much less sexual assault. Following their arrival, irregular migrants had to go through a regulation (legalization) process in Colombia, with 48.1% having either initiated or completed this process. The target migrants have been living in Colombia for an average of 4.3 years, mostly in poor shelters, just as their Colombian neighbours (64.8% and 53% respectively). Both groups, but particularly the migrants, belonged to the lower socioeconomic strata according to their educational levels and occupations (street vendors including street musicians and entertainers; 21.6% of migrants and 10.6% of Colombian neighbours). They all had stressful life events recently, with economic hardship being the most significant one, especially among migrants. In terms of exposure to physical violence, the rates were almost equal for both groups. However, specific incidents such as theft, assault and intended homicide were more often experienced by the Colombian receptor population. Legal services were often sought by migrants, mostly in a special advice centre for migrants or from international organizations while their Colombian neighbours resorted mainly to national institutions including the police. For health issues migrants had often to go to private services with out-of-pocket payment while the Colombians accessed the health system through the subsidized or pre-paid health insurance. Emotional and psychological symptoms were frequent in both groups but more among migrants. The Colombian respondents had more frequently negative comments about migrants but acknowledged that they were exploited in Colombia and that they contribute to community activities and the local economy.

**Conclusion:**

Both Venezuelan migrants and their Colombian neighbours suffer significantly from the consequences of poverty and stressful living conditions. As such, they require equal attention from policymakers and public services. This attention should encompass enhanced security in the public spaces, accessible legal advice, and comprehensive health support.

**Supplementary Information:**

The online version contains supplementary material available at 10.1186/s12889-024-21222-0.

## Introduction

Cross border as well as in-country migration is a global issue. According to current estimations there were in 2020 about 281 million international migrants, which corresponds to 3.6% of the world population [1]. These numbers give only a superficial glance of the daily suffering of a considerable proportion of the population on our planet. Causes of migration are often poor socio-economic conditions, war, violence, and others. Latin America and the Caribbean are experiencing significant changes in migration patterns [2]. Approximately eighty percent of migrants in South America are intraregional migrants [1]. Approximately 52 million people live in Colombia, 2% of whom are Venezuelan migrants. By 2022 Colombia had welcomed around 2.4 million Venezuelans fleeing their homeland [3].

For many decades, Colombia has had severe internal armed conflicts. This internal violence pushed many inhabitants to leave their homes mainly in rural areas and become internally displaced persons (IDPs) in towns and cities [4]. Even for long-ago displacement, the emotional state of those who were displaced remains influential on them, causing mental health issues [5].

Healthcare provision and protection of migrants and IDPs are considered basic human rights. Colombia’s response to regularize Venezuelan immigrants complies with the international human rights law [6]. Many humanitarian NGOs and international organizations are supporting the Colombian government to mitigate the effects of migration. However, there remain significant hurdles to Venezuelan migrants' right to access healthcare in Colombia, often due to a lack of awareness and knowledge of both service users and providers but also to the restrictions for migrants to use general health services with the exception of mother-and-child services which are open to everybody [7]. The conditions and access process need to be better communicated and socialized to meet the needs of migrants [8].

This study was designed to gain a deeper understanding of the mental health [9–12] and social justice needs of Venezuelan migrants in the Colombian border area but also of their Colombian neighbours who may have similar health needs. It particularly focuses on those migrants who intend to, or are forced to, stay there long-term, potentially for years or even indefinitely. Similarly, the Colombian recipient population (“comunidad de acogida" or “welcoming community” called in this paper “receptor population” or “receptors” or “Colombian neighbours”) in the border area, particularly those living near migrants, also exhibit specific health and social justice needs. This situation appears to be typical in areas experiencing a massive influx of people from another country. Therefore, the conclusions of our study hold significant relevance for the ongoing debate on a better management of migration flows.

## Study sites and methods

**Fig. 1:**
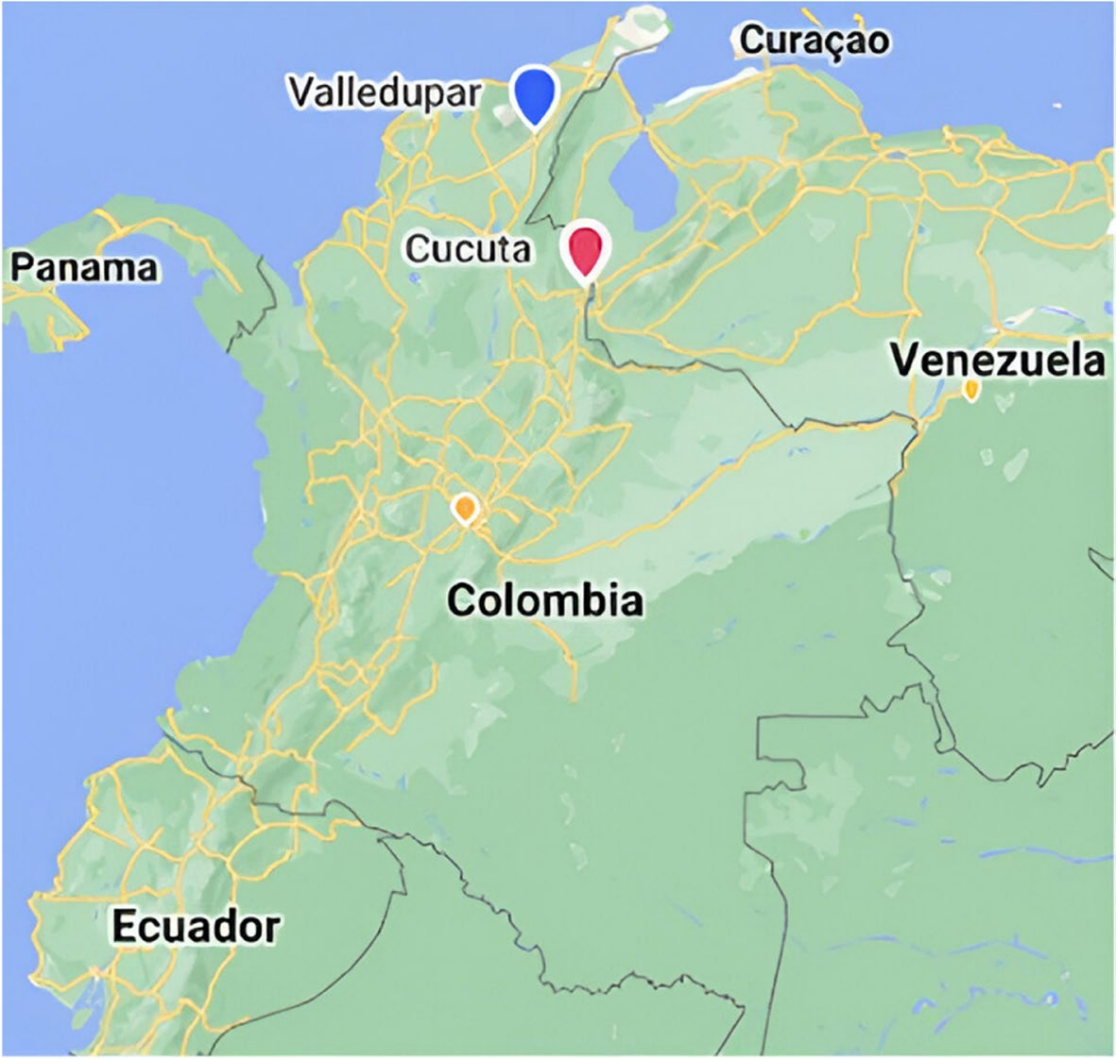
Valledupar and Cucuta (based on my.atlist.com https://my.atlist.com/)

This study was conducted in October/November 2022 in the Colombian cities of Valledupar and Cucuta (see Fig. 1). Valledupar has 483,250 inhabitants [13] and is in the department (State) of Cesar in the Caribbean region. Cucuta with an estimated population of 791,986 inhabitants [14] is in the neighbouring State of Norte de Santander and has in the northeast the border with Venezuela. These two cities were selected for this study given the high inflow of Venezuelan migrants and the progress made by two local partner Universities (Universidad Francisco de Paula Santander in Cucuta and Universidad Popular del Cesar in Valledupar) working on migration issues related to the health and the justice sector.

The climate in both cities is tropical with a mean annual temperature of approximately 30 Centigrade and an average humidity of 56% in Cucuta and 66% in Valledupar (https://weatherspark.com). Commercial activities are the main source of income in both cities.

Near Cucuta lies the primary Colombian-Venezuelan inter-country crossing, where a large proportion of regular migrants (those with the necessary documentation) pass though. In contrast, irregular migrants, lacking the required documents and often without passports, frequently use “trochas” – natural pathways that commonly cross rivers and are notorious for criminal attacks on the migrants. Valledupar lacks an official migration pathway. Regular migrants typically cross the border in La Guajira, the adjacent State to the north, while irregular migrants often use various trochas to enter Colombia. In 2020, 55.9% of migrants were irregular and 44.1% were regular (FND, 2020). Additionally, there was a large number of pendular migrants (4.98 million with permission, tarjeta de movilidad fronteriza, TMF; FND, 2020).

### The study sample

To determine the appropriate sample size [15] for estimating the proportion of respondents with a high number of emotional symptoms (> 6 symptoms per person in the SRQ questionnaire, see below) among migrants and the Colombian population within a 5% margin of error and a confidence level of 95% (95%CI), we took as an estimate for the proportion from our previous study on displaced and non-displaced persons in Valledupar [5]; the respective proportions were 40% among displaced and 20% among non-displaced persons. With a population size of 500,000 and 800,000 respectively we got a minimum sample size for migrants of 369 interviews and for their Colombian neighbours of 246 interviews. This was increased to 412 interviews among migrants and 317 among receptors to allow the analysis of sub-groups.

The inclusion criteria for migrants in our study required them to have been in Colombia for longer than six months. This was to exclude “pendular migrants”, who frequently cross the border for specific services like vaccination, or medication for chronic diseases (according to the statistics of local health services), school education (according to school statistics), and those who stay briefly before moving to other cities or countries staying often in special shelters (called albergues). Our study´s sample included only the more stable migrants drawn from five neighbourhoods in Cucuta and three in Valledupar, each known for having a large migrant population. The number of interviews to be conducted in migrant families of each neighbourhood was proportional to the population size of migrant families in these neighbourhoods, based on the data collected by the local branch of UNHCR (United Nations High Commissioner for Refugees, personal communication). The Colombian receptor families interviewed were those living in the closest vicinity of the migrant families. Consequently, the sample representing the receptor population in our study was not representative of the general Colombian population. Instead, it specifically focused on Colombian families living in close proximity to migrants. For the quasi random selection of families to be interviewed systematic sampling was employed with a sampling interval of 7 (i.e. every seventh household in the neighbourhoods) in Cucuta and 6 in Valledupar calculated by dividing the total number of migrant families by the numbers of interviews to be done. In case this was not feasible, replacement rules were established (e.g. next family to the right if there was a long term absence of the sample family) to avoid any kind of selection bias. Of each migrant family the next Colombian receptor family was chosen for the interview. The non-response rate was < 10% among migrants and around 12% among the Colombian receptor population [5].

Interviewers were local public health officers and officers in the justice system, all of them involved with migration issues in their work. They participated in a training programme on analysing and managing the migration issues. They had never visited before the settlements of migrants and of the Colombian receptor population but were keen to learn more about their living conditions. The preparatory phase of the interview survey included first a training of interviewers about how to deal with traumatic events conducted by two psychologists. The first practical step was contacting community leaders to get their support and to announce the dates of their visit. During this pilot phase, we conducted test interviews, followed by open discussions to assess and validate the questions. This process led to the modification of some questions that were initially unclear [16]. The mental health and violence-related questions were adopted from the Colombian National Interview Survey on Mental Health [17] ensuring their validity and relevance.

The questionnaire in our study (see English translation in the Additional file 1) aimed to gather information on various aspects: the characterization of the study populations and the respondents, their experiences during migration, the receptor population´s perception of migrants, experience of violence and emotional hardships in both groups, and to the use of medical services and the justice system. For self-assessment by the respondents, we included the standardized and internationally validated Self reporting Questionnaire [18, 19]. This tool has also been utilized by the Colombian National Survey [17]. The SRQ has 25 questions on various symptoms (such as headache, sleeplessness, sadness and others). The final three questions, focusing on convulsions, hearing voices and strange external interferences, are specifically aimed at identifying severe mental health conditions.

After identifying the main respondent, who was often the female head of household (either as the responsible person herself or in cases when her husband was away), we proceeded with the study´s procedures. Persons below the age of 18 were excluded from this selection. Before starting the interviews, we explained the purpose of the study and the interviewees’ rights. Subsequently, the respondents were required to sign a consent form.

For the data analysis, we used the “R” open-access software package. Our approach included descriptive statistics and conducting statistical significance testing to assess the findings.

### Ethical approval

Respondents were informed about the procedure, the objectives of the study, the participating institutions and about their right to reject or to finish at any stage the interview without any disadvantage. The forms were anonymous, and no names were recorded. The respondents signed an Informed Consent form. Ethical approval was obtained from Freiburg University and from the two above mentioned participating Colombian Universities (approval on 07.04.2021). Consent for publication was obtained from the persons appearing on the photos.

## Results

### Sample size and characteristics of the respondents

In our study, we interviewed a total of 729 families, comprising 412 migrant families and 317 Colombian receptor families The number of individuals in the sample was 2733 persons, with 1656 migrants and 1077 Colombian neighbours. Among our main respondents, adult women above 18 years of age represented 77.2%, and men accounted for 22.8%. The predominance of women interviewees was probably due to the fact that a large number of interviews occurred when the husband was away (e.g. for leisure activities during the weekend or during working hours on ordinary days) or women were more open to answer a survey; the advantage was that women are considered to be more knowledgeable about family health issues than men [20].

### General characteristics of the study populations and living conditions

Our sample represented the more stable migrant population in the Colombian border area, explicitly excluding pendular migrants who frequently cross the border for medical or educational reasons (as mentioned previously), and those with only a short stay in the area. Additionally, the Colombian recipient population in our study was defined as those living in close proximity to migrants, specifically in neighbourhoods with a high concentration of migrants. The migrants interviewed in our study had been in Colombia for an average of 4.3 years (95%CI 4.1–4.5).

Table 1 presents some characteristics of the two study populations. The slightly lower number of household interviews in the recipient population is attributed to the anticipated greater homogeneity within this group compared to the migrant population. The proportion of males and females in both study populations (migrants and Colombians) was practically the same. The mean age was lower in migrants compared to their Colombian neighbours (22.0 and 28.7 years respectively). Concerning the age distribution there was a larger proportion of minors under 18 years of age among the migrants (47.6%) compared to the Colombian receptor population (36.2%). The other way round, there was a larger proportion of people older than 50 years among the Colombian receptors (18.9%) compared to the migrant population (7.3%). Among those who were interviewed (the respondents) the mean age was 37.0 years (95%CI 36.0–38.1), 34.8 years among migrants (95%CI 33.6–36.2) and 40.1 years (CI 38.2–41.9) among their Colombian neighbours.

**Table 1:** Characteristics of the study population

	Migrants			Receptors		
	n	(%)	95% CI	n	(%)	95% CI
No. family interviews	412	100%		317	100	
Survey population	1656	100		1077	100	
**Average persons / household**	4.03		3.86-4.19	3.38		3.21-3.60
**Gender of the study population:**	n	(%)	95% CI	n	(%)	95% CI
Male	750	45.3	42.9-47.7	509	47.3	44.2-50.3
Female	906	54.7	52.3-57.1	568	52.7	49.7-55.8
Total	944	100		746	100	
**Average age (years)**	22.04		21.26-22.89	28.69		27.55-29.89
**Educational level (≥ 15 years old)**	n	(%)	95% CI	n	(%)	95% CI
None	68	7.2	5.7-9.1	56	7.6	5.8-9.7
Elementary/ primary	327	34.7	31.6-37.8	252	34.4	30.4-37.3
Secondary school	482	51.2	47.8-54.3	336	45.8	41.4-48.7
Superior (college, university)	65	6.9	5.4-8.7	89	12.1	9.7-14.5
Total	942	100		733	100	
**Original Occupation (≥ 15 years old)**	n	(%)	95% CI	n	(%)	95% CI
Student	85	9.1	7.3-11.1	90	12.3	9.9-14.7
Domestic work	354	38.1	34.4-40.7	256	34.9	30.9-37.9
Technician	39	4.2	3.0-5.7	50	6.8	5.1-8.8
Professional	26	2.8	1.8-4.1	31	4.2	2.9-5.9
Others	425	45.7	41.8-48.3	306	41.7	37.5-44.7
Total	929	100		733	100	
**Current occupation (≥ 15years old)**	n	(%)	95% CI	n	(%)	95% CI
Student	66	7.1	5.5-8.9	89	12.1	9.7-14.5
Homework	340	36.6	33.0-39.2	256	34.7	30.9-37.9
Business	37	4.0	2.8-5.4	46	6.2	4.6-8.2
Activities at traffic lights, street musician, street dance, recycler	129	13.9	11.6-16.1	51	6.9	5.2-9.0
Street vendor	71	7.7	6.0-9.4	27	3.7	2.4-5.3
Construction, gardening, agriculture	92	9.9	8.0-11.9	51	6.9	5.2-9.0
Various others	193	20.8	17.9-23.2	217	29.4	25.9-32.5
Total	928	100		737	100	

The educational level (only of persons above 15 years of age) was almost the same in the migrant community compared to the recipient population only that the superior education was low in both groups but much less among migrants (6.9%) compared to the Colombian neighbours (12.1%). Regarding the original occupation (what people used to do in the pre-migration period) there were no major differences between the two groups but when asked for the current occupation there were significant differences particularly in the low income sector and occasional work: 21.6% of migrants were working “on the street” as street musicians, rubbish collectors, or cleaning car windows at traffic lights, or as street vendors. In the Colombian receptor population only 10.6% belonged to this group (*p* < 0.001).

**Fig. 2:**
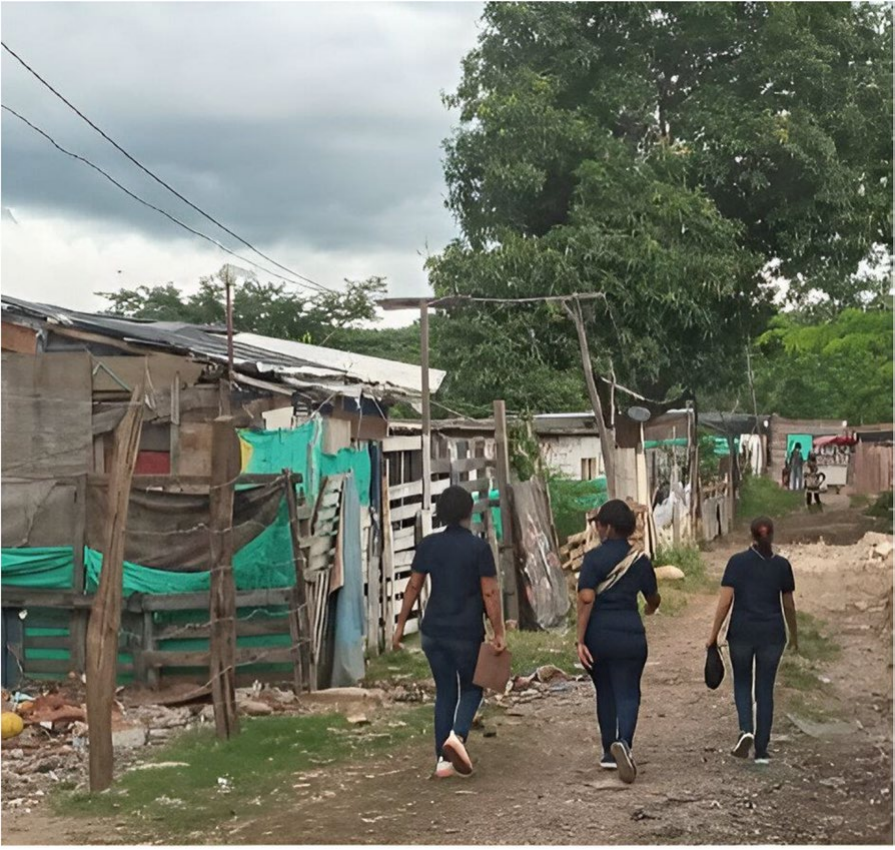
Precarious housing of Venezuelan migrants

**Fig. 3:**
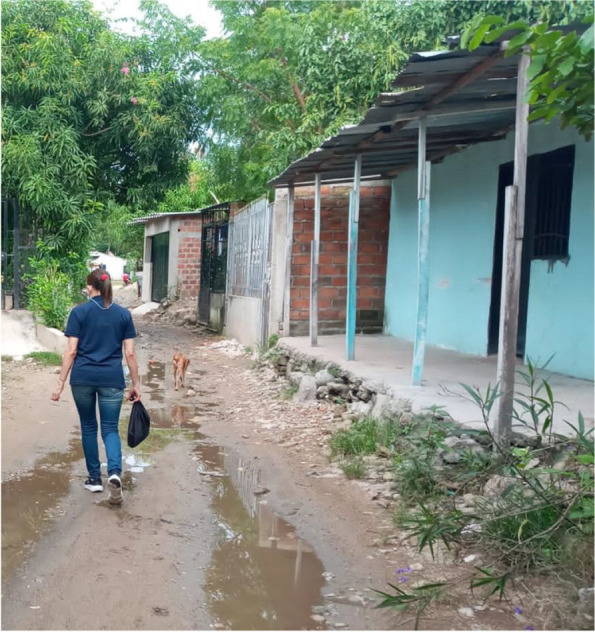
Colombian neighbours of Venezuelan migrants (“receptor population”)

**Fig. 4:**
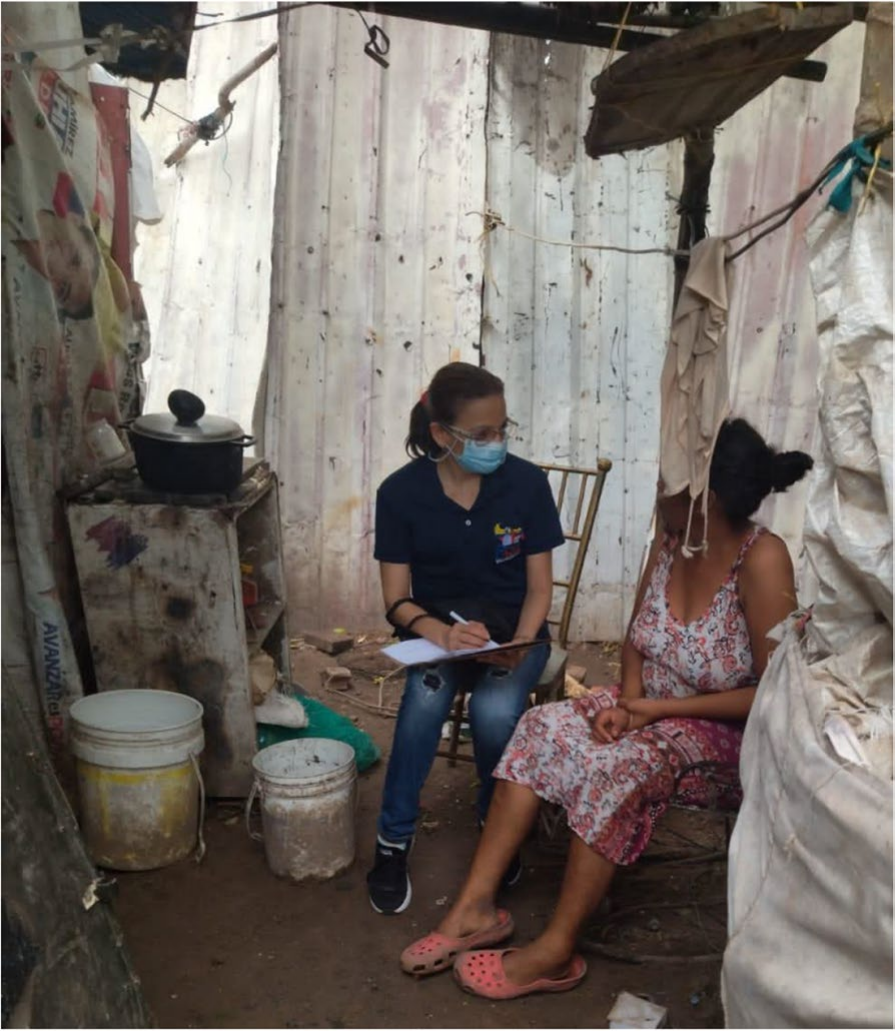
Interview in a migrant household

The living conditions (refer to Figs. 2, 3 and 4) of families interviewed reveal that most lived in informal settlements (“asentamientos”), with 67.2% of migrants and 53.0% of the Colombian receptor population. The remaining families lived in formal neighbourhoods (“barrios”). This is influenced by the sample selection, particularly the receptor families defined as those living close to the migrants. However, the disparity in housing conditions between Venezuelan migrants and their Colombian neighbours was significant: 64.8% of migrant families inhabited precarious houses (ranchos lacking water supply and electricity) compared to 37.5% of the Colombian neighbours (*p* < 0.001). Crowding was similar among migrants (4.03 persons per household) compared to the Colombian neighbours (3.38 per household). A marked difference was observed within the migrant group based on legal status: 42.9% of irregular migrants (those without the permission PPT to stay) resided in precarious shelters (ranchos, cambuches, tiendas) and 43.0% in informal settlements, whereas a smaller proportion (25.7%, *p* < 0.05) of regular migrants (with PPT) lived in such poor conditions and 25.4% in informal settlements.

## Photos 1–3 about living conditions

### Motives and process of migration

Most migrants had left their country for economic reasons (86.4%) and there was no major difference between those arriving at Cucuta (81.7%) and Valledupar (86.1%; *p* > 0.05). Only a few migrated for political reasons (7.4% in Cucuta and 2.5% in Valledupar) and very few because of health issues (3.8%). (NB, the overuse of mother-and-child services and chronic diseases in both cities was mainly caused by pendular migrants who were not part of our sample).

In the Cucuta area, half of the migrants (50.3%) entered Colombia through the regular crossing (bridge of freedom) while the other half entered through informal trochas (see above). In contrast, in Valledupar where there is no official border crossing, most migrants (72.5%) entered through informal trochas. The remaining used the regular pathway in the neighbouring state of La Guajira. Among those who entered via trocha, 48.1% had either received or were in the process of obtaining legal permissions, specifically the Special Permission for Staying (Permiso Especial de Permanencia, PEP) or the Special Permission for Temporary Protection (Permiso por Proteccion Temporal, PPT). Regular migrants predominantly used PEP or PPT for documentation, with 84.4% in Valledupar and 65.3% in Cucuta. In contrast, the use of a Venezuelan identity card or the passport was relatively infrequent.

Almost all migrants (92.1%) had left family members behind in Venezuela, and a considerable proportion (37.0%) continued to transfer money on a regular basis to their families remaining in Venezuela.

### Violence during the migration process

**Fig. 5:**
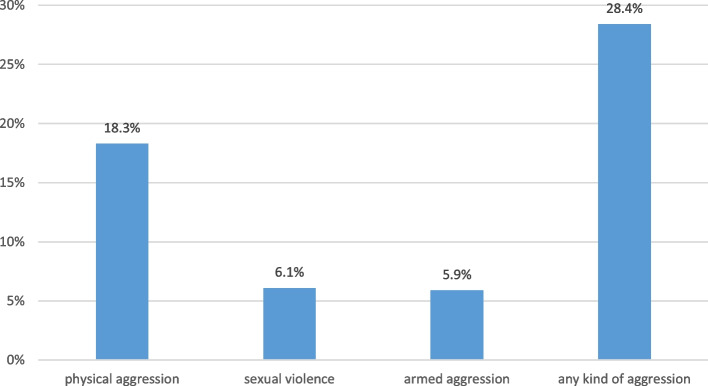
Violence experienced by migrants when crossing the border by an irregular route (trochas). (NB. The respondent could agree to one or more violent events such as physical aggression and sexual abuse)

Regular migrants, crossing the border via official pathways, did not face violence. In contrast, many irregular migrants who entered through trochas experienced violence during their migration process. This was more prevalent in the Cucuta area, affecting 33.2% of migrants, compared to 23.6% in the Valledupar area (23.6%). Among these incidents, physical aggression was the most frequent act (18.3% of all migrants), followed by sexual violence (6.1%) and armed aggressions (5.9%, see Fig. 5 and details below).

### Violence experienced by migrants after arrival and by the receptor population in the Colombian border area

**Fig. 6:**
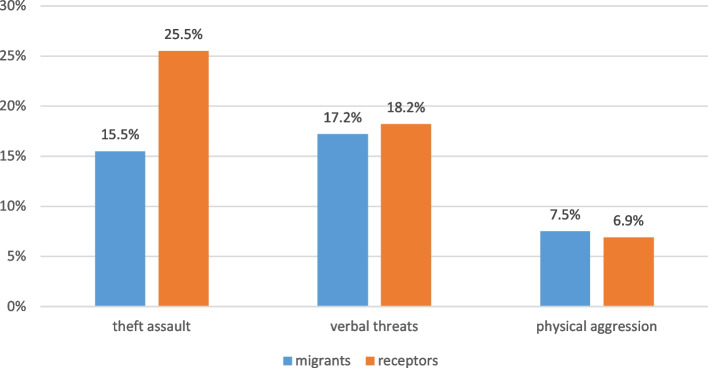
Violence experienced during the preceding year by migrants (*n*= 412) and Colombian neighbors (*n*= 317)

In our study, family members were asked whether they had experienced any of the following in the past 12 months: intent of murder, verbal aggression, theft, burglary, armed threat, physical aggression and/or sexual violence (as detailed in Fig. 6). The most frequently reported were theft and assault, particularly among the Colombian receptor population (25.5% among receptors and 15.0% among migrants; *p* < 0,000). Receiving verbal threats was equally frequent among migrants and their Colombian neighbours (17.2% migrants and 18.9% receptor population; *p* = 0.7). Also, physical aggression suffered by migrants and receptor population were almost the same (7.5% migrants and 6.9% Colombian neighbours; *p* = 0.6). Sexual violence was rarely admitted in both groups (1.7% among migrants and 2.5% among their Colombian neighbours; *p* = 0.6). Intended homicide was more frequently suffered by the Colombian receptor population (7.6%) than by the Venezuelan migrants (3.1%; *p* = 0.01).

Regarding the mental impact of violence, both migrants and their Colombian neighbours had likewise suffered emotional problems (30.8% and 28.4%; *p* > 0.8). They also had after the act of violence difficulties to sleep and bad dreams (23.5% migrants and 19.2% Colombian neighbours; *p* = 0.2) while migrants hade significantly more symptoms of heart beating, sweating, and sleeping disorders (20.9%) than their Colombian neighbours (13.9%; *p* = 0.03).

**Fig. 7:**
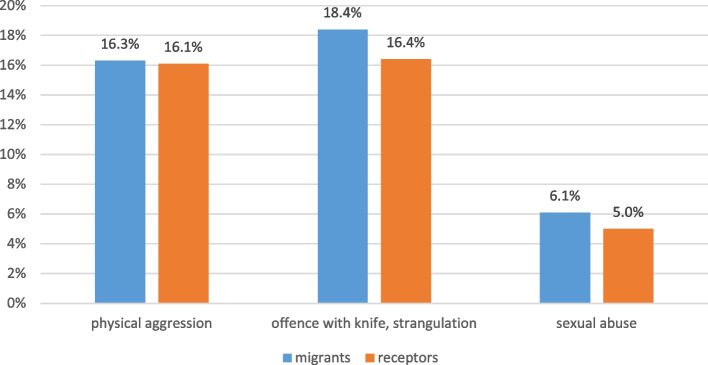
Intra-domestic violence among migrants (412) and Colombian receptor population (*n*=317). (NB. The respondent could agree to one or more violent events such as physical aggression and sexual abuse)

Regarding intra-domestic violence (Fig. 7), there were no differences between the two groups: 16.3% in migrant families and 16.1% in Colombian receptor families had suffered from aggressions like receiving kicks, bites and hits. Even more severe offences were reported in both groups (18.4% in migrant families; 16.4% in Colombian families) such as attack with a knife or another weapon, burning or strangulation. Intra-domestic sexual abuse was reported by 6.1% migrant families and 5.0% Colombian neighbours.

### Other stressful events during the past 12 months

The predominant concern for both groups was economic challenges, with 69.4% of migrants and 59.9% of their Colombian neighbours (*p* = 0.007). Another significant difference was in adapting to major life changes; 34.0% of migrants versus 23.0% of their Colombian neighbours had to deal with events such as a new spouse, a new job, pregnancy or a new-born baby (*p* = 0.002). Other common stressors were similar between the two groups: job loss (35.9% migrants; 32.5% receptors), severe health problems (31.1% migrants; 30.6% Colombians), severe sickness of a close relative (46.6% migrants; 41.3% Colombians) or even death of the spouse or a close friend or colleague (5.8% migrants; 4.1% Colombians).

### Use of legal services by migrants and receptor population

Migrants primarily sought legal advice from the special migration office (22.9%), followed by international cooperation organizations like the IOM, UNHCR, GIZ and others (18.5%). They also approached the private legal sector (17.1%) or even the police (13.5%). In contrast, the recipient population more frequently consulted the Colombian Institute for Family Welfare (Instituto Colombiano de Bienestar Familiar, ICBF, 23.9%), the police (19.9%) or the legal services (12.4%). When asked about the resolution of their legal problems, migrants reported the highest success rate with the migration office (24.5% of cases solved). In contrast, for their Colombian neighbours, the most effective sources were the police (21.7%) and assistance from friends (21.3%).

### Use of health services by migrants and receptor population

Over the past 12 months, a significant proportion of families in both groups visited general health services, with 72.6% of migrant families and 73.5% of receptor families reporting at least one member who did so. The use of mental health services was much less common: 15.0% of migrant families and 15.8% of receptor families used these services in the same period. Migrants used different services for solving their health problems but mainly the subsidized services (hospitals, health centres and others: 43.4%) or they asked friends or family members for advice (12.9%) or bought medicine in pharmacies (23%). In contrast, the recipient population went almost exclusively to the formal health services (87.2%) and went rarely directly to pharmacies for medicine (9.4%). On average, migrant families utilized mental health services 2.44 times (95%CI 1.98–3.0), while the receptor population used these services slightly more (2.78 times per family; 95%CI 2.18–3.70). The use of general health services was the same in both groups (3.5 times in 12 months; 95%CI 3.25–3.85).

### Opinions of Colombian residents (“receptors”) about Venezuelan migrants

**Fig. 8:**
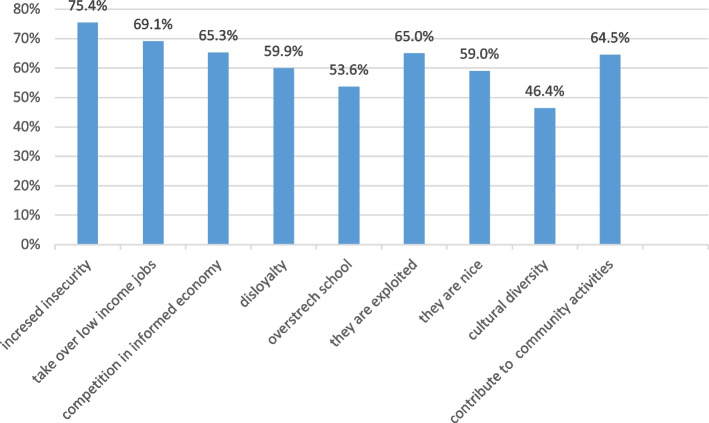
Opinions of Colombian residents (receptors, *n*=317) about migrants

The local Colombian residents had more negative than positive opinions about Venezuelan migrants (Fig. 8): 75.4% thought that the arrival of migrants increased insecurity and delinquency. Additionally, 69.1% felt that migrants were taking over jobs in the low-income sector, and 65.3% held the view that migrants competed heavily in the informal economic sector with Colombians. Migrants were perceived by 59.9% of Colombian respondents of being disloyal and 53.6% thought that the children of migrants are prioritized to fill the limited quota for entering school. It was, however, also acknowledged by 65% that the labour force of migrants suffered of heavy exploitation by their employer. Furthermore, it was found to be unjust that international aid organizations worked exclusively for migrants and not for the poor local population.

There were also positive feelings about migrants: 59.0% reported that migrants in general are nice people and that they contribute to the cultural diversity (music, dancing, dishes; 46.4%) and to product diversity (34.4%). Additionally, 44.5% recognized migrants’ active participation in community activities and their contribution to the local economy (23.0%).

### Emotional and psychological symptoms experienced by the respondents during the previous 30 days

In our study, individual responses were gathered using the internationally validated Self Reporting Questionnaire [18], which had also been applied in the Colombian National Mental Health Survey [17]. The results indicated that Venezuelan migrants experienced certain symptoms more frequently than their Colombian neighbours (*p* < 0.001). These included sleeplessness (49.0% among migrants vs. 35.6% among Colombians), fright (54.4% vs. 41.0%) and tiredness (39.8% vs. 27.1%). Other symptoms were less frequent among migrants and more among the Colombian recipient population (e.g. suicidal thoughts (10.9% versus 12.3%), or loss of interest (20.4% versus 24.9%) but the differences were not statistically significant (*p* > 0.05).

**Fig. 9:**
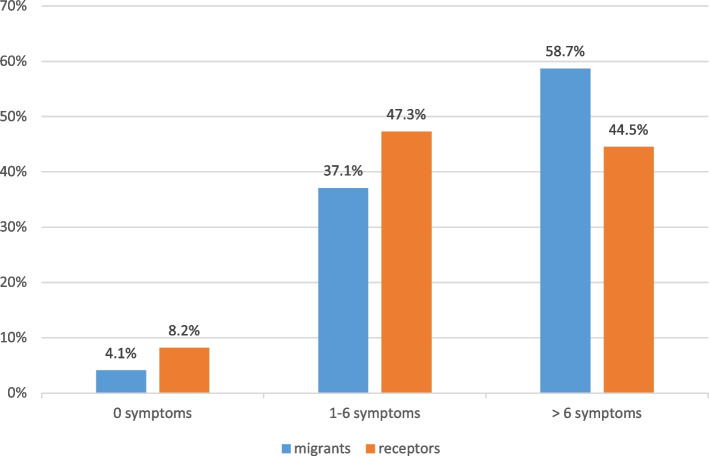
Number of psychosomatic symptoms experienced by respondents (migrants and Colombian receptor population) during the preceding 3 months

The overall picture becomes clearer when grouping the study population into a) respondents with no symptoms, b) respondents with a few symptoms (2 to 6) and c) respondents with many symptoms. Figure 9 shows that among the migrants the proportion with many symptoms was predominant (58.7% had more than 6 symptoms as opposed to the Colombian recipients who had significantly less symptoms (44.5% with more than 6 symptoms; *p* < 0.03). The recipients had either no symptoms (8.2%) or few symptoms (47.3%) while the migrants had either no symptoms (4.1%) or only a few (47.3%). The differences between migrants and their Colombian neighbours were all statistically significant (*p* = 0.03–0.0002).

## Discussion

Migrants were on average younger than their Colombian neighbours (22 vs 29 years). Both groups belonged mostly to the lower socio-economic population strata which is different from previous waves of the Venezuelan migration towards Colombia during the early days of the Venezuelan socialist rule when mainly intellectuals and business people fled the country [21]. Although both migrants and receptor population live under difficult conditions, the migrants –particularly the irregular ones- have to face significantly more often poor housing in provisional shelters or tents and stay in informal settlements with no electricity and piped water compared to the receptor population (43% versus 25%). Migrants also have to do unpleasant work “on the street” particularly at traffic lights to get a small donation.

Roughly half of the migrant families came to Colombia through the regular route and the other half arrived irregularly through informal pathways (trochas). Half of the irregular migrants (48.1%) were in the process of legalization (PPT), in other words, they were no criminalized asylum seekers [22] like in most countries of the European Union where particularly children with an irregular status were exposed to the risk of poverty, exploitation discrimination and social exclusion [23]. The Colombian protection laws of migrants (providing the opportunity for irregular migrants to stay in the country) is based on international regulations for human rights and much less restrictive than migration laws in many high-income countries. However, migrants did not receive sufficient financial support from the Colombian government, had to care for themselves leading them often to live a life in poverty and were exposed to xenophobia, a phenomenon also well known in the European Union [22].

There were several stress factors for the migrants:

i) Irregular border crossing through trochas meant a risk of being exposed to violence which occurred in 33.2% of crossings in Cucuta, slightly less in Valledupar being physical aggression the most frequent one (18.3%).
ii) After the arrival in Colombia the migrants suffered other stress factors like living in precarious shelters with no electricity and water supply as well as leaving family members behind (92.1%) and having to regularly transfer money to them (37.0%).
iii) Facing financial problems was frequent (69.4%) as well as change of job or family issues.
iv) Being exposed to xenophobia or to exploitation by landlords or employers was mentioned in the interviews of Colombian receptor families.
v) The risk of assaults and theft was mentioned based on previous experiences (15%).
vi) Almost 20% of migrants (19.3%) had to work on the street mainly at traffic lights cleaning car windows or showing acrobatics or they worked as street vendors or in other areas of the informal economic sector.
vii) While regular migrants could use mostly the subsidized public health services the irregular migrants could rarely access the Colombian health system and most of them had to pay “out-of-pocket” for private services [24].

The Colombian neighbours were also exposed to these issues but less frequently. Particularly financial problems (59.9%) and important life changes (new job, new spouse, and others) were frequent but also work at traffic lights (12%) as described above. Regarding criminal acts the Colombians suffered more frequently (25.4%) than the migrants. This is in line with what we found in an analysis of the criminal statistics: The relative risk of committing a crime (persons above 14 years of age) was significantly higher for Venezuelan migrants than for the Colombian population particularly for shop lifting (RR = 5.6), theft (RR = 2.1) and drug trafficking (RR = 2.9; unpublished) [25] illustrating the harsh living conditions particularly of migrants.

The stress factors described above resulted in a considerable number of psychosocial symptoms (> 6 per person), significantly more frequent among migrants (58.7%) but also frequent among their Colombian neighbours (44.5%) which may result in post-traumatic stress disorder (PTSD) [26]. Severe mental health issues such as suicidal thoughts or convulsions were rare in both groups. The use of mental health services (which is influenced by access and behavioural factors [27], was almost the same for migrants and receptors (on average 2.6 visits during the previous year) as was the use of general health services (3.5 visits per person per year).

Regarding legal services, the availability of a special migration office and support services by international organizations such as IOM (International Organization of Migration) was crucial for migrants to get legal advice. However, no detailed analysis of the accessibility of legal services could be done. For this, criteria related to institutional barriers (trust, information, efficiency, effectiveness, formalism, and bureaucracy) social, cultural, and economic barriers (economic, geographical, linguistic and gender) have to be considered [28]. The Colombian receptor population resorted for legal advice more to the Colombian Institute for Family Welfare.

### Recommendations

In a short seminar at the end of the study the following recommendations were mentioned by participants, including a number of migrants: Mitigation strategies dealing with the hardships of migrants and their Colombian neighbours should include offering capacity building for productive work like urban gardening, bakeries, and other options for creating additional income for families and improving food security. Also, self-help in construction work for improved housing should be discussed. Often leisure activities will help to bring different people together and reduce social tensions like women’s clubs or sport activities for the youth [29]. Strengthening primary (non-specialized) low-cost health care including mental health care as recommended by OIM and others [30] should be considered and organized together with the academic sector working on Primary Care.

### Limitations of the study

As mental health issues are delicate and not easily admitted in a formal interview, interviewers (predominantly females) were thoroughly trained to deal with emotional reactions but also to give time to respondents when asking sensitive questions. In the pilot phase when we combined qualitative and quantitative interviews, we could ascertain the agreement between interview responses and subsequent in-depth interviews. Also, the relatively low non-response rate of roughly 10% shows that it was possible to establish with the help of community leaders and other authorities confidence with the interviewers allowing them to ask some sensitive questions about violence and other issues. Questions on alcohol abuse and drug consumption were excluded which would have required a different survey approach.

Additionally, the quantitative survey was complemented by a qualitative study which reveals people’s feelings and experiences and will be published in a separate paper.

## Conclusions

- The legal system regulating the rights and obligations of regular and irregular (undocumented) migrants and providing (until November 2023) a fast track towards legalization of irregular migration provides the motivation for irregular migrants to get legalized and thus access to the social security system.
- Insufficient public support for migrants but also for their Colombian neighbours who live under similar conditions, contributes to high levels of poverty and crime rates
- The higher number of stress factors among migrants compared to their Colombian neighbours (who also live under remarkable constraints) adds to the usual challenges posed by the daily struggle for survival.
- Considering the fact that migrants are younger than their Colombian neighbours and have taken the important decision to leave their country may strengthen their resilience to dealing with difficult living conditions
- The fight against xenophobia and comprehensive information of the Colombian public about the hardships migrants and also their Colombian neighbours are facing may help to decrease tensions, favour social integration and protect mental health
- Creating new job opportunities for migrants and the Colombian receptor population may potentially reduce the crime rate and strengthen community networks.

## Supplementary Information

Supplementary Material 1.

## Data Availability

All original data are available in "R" format from the corresponding author.
