# Supplementary material for: Facing migration under adverse conditions: challenges and resilience in the Colombo-Venezuelan border area
Source: BMC Public Health. 2025 Jan 22;25:261. doi: 10.1186/s12889-024-21222-0 (PMC11752629; doi:10.1186/s12889-024-21222-0)
Supplement: Supplementary file 1 — Supplementary Material 1. [file 12889_2024_21222_MOESM1_ESM.docx]

**Additional file 1. Study questionnaire (English translation)**

**Survey´s Number: ­______**

**MENTAL HEALTH SURVEY AMONG MIGRANT POPULATION AND HOST COMMUNITY.**

**INTRODUCTION TO THE SURVEY:**

This survey contributes to the objective defined by the PAGEL project to determine the burden of mental disorders and risk factors among migrants and the host population. The study is supported by the territorial health authority and other national and international institutions. Your participation is voluntary, and you are free to respond or not to any of the questions. This survey is anonymous; names or addresses are not required. Your participation supports the definition of intervention alternatives to the situation faced by the population undergoing the migration process in relation to the health and justice sector.

1. Are you? 1. Colombian ___2. Venezuelan Migrant___ 3. Returned Colombian___
2. How long have you been living in Colombia? Please indicate:

If you have been in Colombia for less than a year (FROM 1 TO 11 months), How many?__________

If you have been in Colombia for more than a year (FROM 1 TO 10 years), How many?__________

1. What is the location of your home?
2. Settlement___ 2. Neighborhood___ 3. Other: ___ 4. If your answer is Other, please specify:___________________
3. ¿Where do you live in? 1. Room___ 2. Apartament___ 3. House___ 4. Shack___ 5. Makeshift shelter___6. Tent___ 7. Boarding house/hotel___ 8. Other: ___

8. If your answer is Other, please specify___________________________________

1. ¿Who do you live with? (Fill the table including the interviewee)

| **Number of people form 1 to 15 (the interviewee is number 1)** | **Gender**  1=Male  2=Female | **Age**  AGE IN YEARS (MANUALLY RECORD, PLEASE USE “0” FOR UNDER ONE YEAR OLD) | **Education**  0=None  1=Primary  2=Secondary  3=Higher Education/Technicianl | **Original Profession**  1=Student  2=Homemaker  3=Training Technicians  4=Professional training  5=Other occupation | **Current Occupation**  1=Student  2=Home  3=Commercial activities  4= Various jobs at traffic lights, Street musician, delivery man or waste recycler  5= Door-to-door salesman  6.= Construction, gardening or or agriculture.  7=Other |
| --- | --- | --- | --- | --- | --- |
| 1 |  |  |  |  |  |
| 2 |  |  |  |  |  |
| 3 |  |  |  |  |  |
| 4 |  |  |  |  |  |
| 5 |  |  |  |  |  |
| 6 |  |  |  |  |  |
| 7 |  |  |  |  |  |
| 8 |  |  |  |  |  |
| 9 |  |  |  |  |  |
| 10 |  |  |  |  |  |

1. Number of the interviewed person (in the above table) MANUALLY RECORD__________

**NEXT QUESTIONS ARE ONLY FOR COLOMBIANS:** (Host population)

1. How has the arrival if migrants affected your neighborhood?

| **Positive effects** | **1.Yes** | **2.No** | **3.unknown** |
| --- | --- | --- | --- |
| Improves the local economy |  |  |  |
| Diversity in music, dance, and food |  |  |  |
| Active participation in the community |  |  |  |
| They are generally kind |  |  |  |
| Greater diversity of products |  |  |  |
| **Negative effects:** | **1.Yes** | **2.No** | **3.Unknown** |
| Increased unfair competition |  |  |  |
| Displacement of local labor |  |  |  |
| Labor exploitation |  |  |  |
| Increase in security and crime |  |  |  |
| Competition in the informal market |  |  |  |
| Priority in school places |  |  |  |
| Unfair distribution of aid by international cooperatives |  |  |  |

**QUESTIONS 8 TO 14 ARE ONLY FOR VENEZUELAN MIGRANTS AND RETURNED COLOMBIANS.**

1. What were your reasons for leaving Venezuela? 1. Political___2. Economic__ 3. Family___4. Health___ 5. Other: ___6. If Other, please specify ________________(MANUALLY RECORD)
2. Did you live part of your family in Venezuela? 1. Yes ___ 2. No___
3. Do you send remittances to Venezuela from your income? 1.Yes __2. No__ 3. Regularly______
4. Have you processed a regularization document in Colombia (PEP/PPT)?

1. Yes___ 2. No___

1. What has been the regularization document you have processed in Colombia?:
2. Special Stay Permit(PEP)/ Temporary Protection Permit (PPT)___
3. Unique Registry of Venezuelan Migrants (RUMV)
4. Safe-conduct pass___
5. Other__________ if Other, please specify_______ (MANUALLY RECORD)
6. How did you enter Colombia?: 1. Regular crossing___ 2. Unofficial path___
7. Have you or someone in your family been a victim of violence during the migration process? (See table)

| **Types of Violence** | 1. **Yes** | 1. **No** |
| --- | --- | --- |
| Attempted Homicide |  |  |
| Verbal threat |  |  |
| Robbery or mugging |  |  |
| Threat with a ewapon |  |  |
| Physical aggression (Beaten, kicked, pushed) |  |  |
| Sexual violence |  |  |
| Other, please specify__________________________ |  |  |

------------------------------------------------------------------------------------------------------------------------

**NOW LET'S TALK ABOUT VIOLENCE. QUESTIONS FOR MIGRANTS AND COLOMBIAN POPULATION.**

1. ¿Have you someone in your family been a victim of violence in the Colombian territory during the last 12 months? (See table)

| **Types of violence** | 1. **Yes** | 1. **No** |
| --- | --- | --- |
| Attempted Homicide |  |  |
| Verbal threat |  |  |
| Robbery or mugging |  |  |
| Threat with a weapon |  |  |
| Physical aggression (Beaten, kicked, pushed) |  |  |
| Sexual violence |  |  |
| Other, please specify__________________________ |  |  |

**INDIVIDUAL QUESTIONS FOR THE INTERVIEWEE.**

1. If you are a victim, have you experienced emotional issues/anxiety?

1. Yes___ 2. No___

1. Due to stressful violence, have you experienced one or more of the following signs:

| **Signs** | **1. Yes** | **2. No** |
| --- | --- | --- |
| Recurring disturbing thouhts |  |  |
| Disturbing dreams or difficulty sleeping |  |  |
| Reactions such as palpitations, difficulty breathing, or sweating |  |  |
| Avoidance of thinking or talking about the stressful experience |  |  |

1. Have you experienced any of the following events during the las 12 months?

| **Event** | **1.Yes** | **2.No** | **3.No Apply** |
| --- | --- | --- | --- |
| Got divorced or separated |  |  |  |
| Lost your job |  |  |  |
| Had serious health problems |  |  |  |
| Faced severe financial problems |  |  |  |
| Your spouse, partner or companion died |  |  |  |
| A family member (including uncle/aunt) or someone very close to you became ill or died |  |  |  |
| Had a significant change in your life such as a new partner, pregnancy, birth of a child or job change |  |  |  |

1. Symptom Questionnaire (Self-Reporting Questionnaire, SRQ) for the interviewee (En relación a los últimos 30 días).

| **PREGUNTA** | **1.SI** | **2.NO** |
| --- | --- | --- |
| 1. Do you often have headaches? |  |  |
| 2. Do you have a poor appetite? |  |  |
| 3. Do you sleep poorly? |  |  |
| 4. Do you get startled easily? |  |  |
| 5. Do you suffer from hand tremors? |  |  |
| 6. Do you feel nervous, tense or bored? |  |  |
| 7. Do you suffer from poor digestion? |  |  |
| 8. Are you unable to think clearly? |  |  |
| 9. Do you feel sad? |  |  |
| 10. Do you cry very often? |  |  |
| 11. Do you have difficulty enjoying your daily activities? |  |  |
| 12. Do you have difficulty making decisions? |  |  |
| 13. Do you have difficulty doing your work? (Do you struggle with your work?) |  |  |
| 14. Are you unable to play a useful role in your life? |  |  |
| 15. Have you lost interest in things? |  |  |
| 16. Do you feel that you are a worthless person? |  |  |
| 17. Have you had the idea of ending your life? |  |  |
| 18. Do you feel tired all the time? |  |  |
| 19. Do you have unpleasant sensations in your stomach? |  |  |
| 20. Do you get tired easily? |  |  |
| 21. Do you feel that someone has tried to harm you in some way? |  |  |
| 22. Are you a much more important person that others think? |  |  |
| 23. Have you noticed interference or something strange in your thinking? |  |  |
| 24. Do you hear voices without knowing where they come from or that other people cannot hear? |  |  |
| 25. Have you had seizures, attacks, or falls to the ground with movements of arms and legs; with tongue biting or loss of consciousness? |  |  |

**NOW WE WILL TALK ABOUT THE VIOLENCE THAT OCCURS IN MANY FAMILIES.**

1. Have you or someone in your family been: pushed, kicked, bitten, punched, dragged? 1. Yes ____ 2. No_____ 3. No response_____
2. Have you or someone in your family been attacked with a knife or another weapon or been attempted to be strangled or burned? 1. Yes ____ 2. No___ 3. No response _____
3. Have you or someone in your family been a victim of a sexual abuse event in your home? 1. Yes____ 2. No___ 3. No response_____

**NEXT, I WILL ASK YOU SOME QUESTIONS REGARDING HEALTH SERVICES.**

1. How many times have you or someone in your family sought attention in the last 12 months for emotional, anxiety, or mental health issues? ______________________________________________________________

______________________________________________________________

1. How many times have you or someone in your family sought attention in the last 12 months for other health issues?

______________________________________________________________

______________________________________________________________

1. When you sought health services in the last 12 months, were you attended to?
2. Yes ____ 2. No___ 3. No response_____ 4. Does not apply_____

**IF THE ANSWER TO THE PREVIOUS QUESTION WAS: (2. No/3. No response/NA) PLEASE SKIP TO QUESTION No. 27. IF THE ANSWER WAS: 1. YES, CONTINUE WITH QUESTION 26**

1. What were the main reasons for **NOT having** received attention for health problems?

| **Reasons for NOT consulting** | **Mark with an (X)** |
| --- | --- |
| Neglect/It was not necessary/Did not have time |  |
| The insurer did not authorize the care |  |
| They did not give an appointment or they gave it for a far-off date |  |
| Too many procedures were required |  |
| Did not believe they could help |  |
| Did not trust the doctors |  |
| Fear of diagnosis and treatment |  |
| Did not know they had the right to this service |  |
| You went but was not attended to |  |
| Did not have money |  |
| The schedules did not fit |  |
| The care site was too far away |  |
| Lack of knowledge about access routes or care |  |
| Another reason: (MANUALLY RECORD THE REASON) |  |
| Did not need them - Does not apply |  |

1. The last time you or someone in your family needed health care, you went to::
2. Health service (Hospital, Clinic, etc.) ___
3. Drugstore or pharmacy____
4. Alternative medicine (Homeopathic, acupuncture, traditional healers, etc.)____

4. Relatives, friends, colleagues___

5. Health Service exclusive for migrants____

6. Other___

1. Who paid most of the expenses for this service?
   1. Public Health Insurance: Contributory___
   2. Public Health Insurance: Susidized____
   3. Prepaid medicine plan/Private insurance___
   4. With own resources___
   5. No payment was required___
   6. Cooperants/donations___
   7. Other___

**NEXT, WE WILL ASK YOU SOME QUESTIONS ABOUT LEGAL SERVICES IN COLOMBIA.**

Have you or someone in your family approached any of the following entities in the last 12 months to ask for support?

| **Entities** | **Mark with an (X) One or Several options** | **Did they solve your problem? 1. YES -2.NO** |
| --- | --- | --- |
| Police |  |  |
| Colombian Institute of Family Welfare |  |  |
| Migration office |  |  |
| Judge for guardianship |  |  |
| Ombudsman |  |  |
| Family commissariat |  |  |
| Public defender's office |  |  |
| Legal clinic |  |  |
| International cooperants or Non-governmental organization |  |  |
| President of Community Councils |  |  |
| Private individuals or others |  |  |

**THANK YOU VERY MUCH FOR YOUR ATTENTION.**
